# Quality of community basic medical service utilization in urban and suburban areas in Shanghai from 2009 to 2014

**DOI:** 10.1371/journal.pone.0195987

**Published:** 2018-05-23

**Authors:** Lijun Guo, Yong Bao, Jun Ma, Shujun Li, Yuyang Cai, Wei Sun, Qiaohong Liu

**Affiliations:** 1 Shanghai University of Medicine & Health Sciences, College of Health Information Technology and Management, Pudong New District, Shanghai, China; 2 Shanghai Jiao Tong University School of Public Health, Shanghai, China; 3 Hongqiao International Institute of Medicine, Shanghai Jiao Tong University School of Medicine, Changning District, Shanghai, China; 4 Shanghai Tongren Hospital, Changning District, Shanghai, China; 5 Zhengzhou Fifteenth People’s Hospital, Shangjie District, Zhengzhou City, Henan Province, China; University of California San Diego, UNITED STATES

## Abstract

Urban areas usually display better health care services than rural areas, but data about suburban areas in China are lacking. Hence, this cross-sectional study compared the utilization of community basic medical services in Shanghai urban and suburban areas between 2009 and 2014. These data were used to improve the efficiency of community health service utilization and to provide a reference for solving the main health problems of the residents in urban and suburban areas of Shanghai. Using a two-stage random sampling method, questionnaires were completed by 73 community health service centers that were randomly selected from six districts that were also randomly selected from 17 counties in Shanghai. Descriptive statistics, principal component analysis, and forecast analysis were used to complete a gap analysis of basic health services utilization quality between urban and suburban areas. During the 6-year study period, there was an increasing trend toward greater efficiency of basic medical service provision, benefits of basic medical service provision, effectiveness of common chronic disease management, overall satisfaction of community residents, and two-way referral effects. In addition to the implementation effect of hypertension management and two-way referral, the remaining indicators showed a superior effect in urban areas compared with the suburbs (P<0.001). In addition, among the seven principal components, four principal component scores were better in urban areas than in suburban areas (P = <0.001, 0.004, 0.036, and 0.022). The urban comprehensive score also exceeded that of the suburbs (P<0.001). In summary, over the 6-year period, there was a rapidly increasing trend in basic medical service utilization. Comprehensive satisfaction clearly improved as well. Nevertheless, there was an imbalance in health service utilization between urban and suburban areas. There is a need for the health administrative department to address this imbalance between urban and suburban institutions and to provide the required support to underdeveloped areas to improve resident satisfaction.

## Introduction

Many rural areas lack adequate medical centers and trained doctors, particularly specialists, tend to be small in size, and have lower service offer and financial difficulties[1, 2]. A comparative study of health resources between suburban and urban areas showed that urban health departments serving a larger area had more capital and staff, established more partnerships, and provided more health services than those in the suburbs or rural health administrative departments[3]. Community health centers play an important role in the United States, Mexico, and other countries. Importantly, studies showed that imbalances between urban and rural community health services exist in the United States and Mexico[4–6], but data about eventual urban/suburban imbalance are lacking for China.

Community health service centers integrating six functions (including medical treatment, prevention, health care, rehabilitation, health education, and family planning guidance) have become an important part of the medical service system in China, and they are the basic link to achieving primary health care for all[7]. Well conducted urban and suburban community health service work in Shanghai can not only ease medical health service imbalances in urban areas but can also solve the most basic health service needs of suburban residents. This type of health service work is one of the most effective ways to alleviate the burden of suburban residents[8]. Investigations of community health service utilization in China have more frequently focused on community residents[9–14]. Very few reports have focused on urban and suburban health service centers as research objectives to understand the quality of health service utilization. In 2006, Ma Yanan et al.[8] utilized health resource input, configuration, and output data from 24 community health service centers in four urban and suburban districts of Shenyang to evaluate comprehensive and relative efficiency and employee satisfaction. A study conducted by Zhu Meiling et al.[15]examined the efficiency of community health services in urban and suburban areas of Shanghai using community health centers. The Douglas production function method (an econometric method) was used for the comparison of the quality of basic medical service utilization between urban and suburban community health centers. Zhang and Yong[16] also focused on the community health center as their study objective. They applied principal component analysis, although it was solely a factor analysis of public health assessment results in 18 community health centers in Shanghai in 2011, and the quality of basic medical service utilization in urban and suburban areas of Shanghai community health centers was not compared. Therefore, studies about the urban/suburban discrepancies in China are limited and well-conducted studies are necessary to improve our knowledge and improve the health care system in China.

A series of articles have reported that measurement tools such as accumulative synthetic scoring (ASS), the rank sum ratio (RSR), the technique for order preference by similarity to ideal solution (TOPSIS), fuzzy comprehensive evaluation (FUZZY), and analytic hierarchy process (AHP) have been applied to complete the evaluation of community health services since 2002[17–19]. Nevertheless, studies using the principal component analysis to analyze the quality of health service utilization between urban and suburban areas are rare. Therefore, this study focused on the quality of medical service utilization (benefit and effect of medical service quality, overall satisfaction of the residents, and quality of the two-way referral effect) in urban and suburban community health centers in Shanghai between 2009 and 2014. The results could help guide policies for macro-management and scientific decisions regarding community health services in Shanghai.

## Subjects and methods

### Subjects of the study

This cross-sectional survey assessed 73 community health service centers. After consent of the community health centers, the study evaluated data collected over 6years, i.e. from 2009 to 2014. According to the geographical position of the community health centers, the urban group covered Xuhui District, Yangpu District and Jing’an District, and the suburban group covered Jinshan District, Jiading District and Fengxian District.

### Study methods

#### Ethic statement

This study involved no ethical issues. The survey was approved by the 73community health centers prior to participation. All information obtained from the survey was anonymized and de-identified prior to analysis.

#### Survey and sampling methods

Using a two-stage random sampling method, 80 community health service centers were randomly selected from six districts that were also randomly selected from 17 counties in Shanghai. During the survey, one self-designed questionnaire based on routine reports (such as financial statements, human resource reports, and medical statistics reports) from each organization were used by trained investigators who went to the appointed institutions to collect the data and complete the survey. The questionnaire was written in Chinese and consisted of seven parts: service population, revenue and expenditure, human resources, services, overall resident satisfaction, two-way referral, and information (see details in S1 Table). A total of 80 questionnaires were distributed, and 73 valid questionnaires were retrieved (response rate of 91.2%).

### Research contents and relevant indexes

In this survey, the evaluation index for urban and suburban community health centers encompassed the service population index (including the total population, registered household population, people aged 60 years and above, and children under 6 years of age), efficiency indexes (including the number of annual outpatient visits), benefit indexes (including outpatient expenditure per-time and home bed-day costs), common chronic disease management effectiveness indexes (behavior correction rate and control rate of hypertension and diabetes mellitus), overall satisfaction of residents, and two-way referral effectiveness indexes (including cases of referrals to a superior hospital and to an inferior hospital). The indexes were extracted from the reporting forms (including finance, human resources and medical service reporting forms) established by the Shanghai Municipal Health Bureau. These forms are used by all types of institutions and organizations in Shanghai, and the bureau releases a statistic report annually. A registered permanent resident refers to a person who has a registered permanent residence in a regular residence of the Public Security Household Registration Management Authority according to the Regulations on Residence Registration of the People’s Republic of China. This type of population is defined irrespective of whether or for how long its individuals travel; as long as they have a registered permanent residence in a region, they are regarded as the region’s household population.

### Statistical methods

The database was established and managed using Epidata 3.0. Statistical analyses were performed using SPSS 19.0. Levene’s test was used to analyze the normality of the continuous data. Normally distributed variables were analyzed using the t-test for independent samples or a one-way analysis of variance. Non-normally distributed variables were analyzed using a non-parametric test. Categorical data were described by ratios and differences among groups were analyzed using the chi-square test. Each future indicator was predicted using Eviews8.0. SPSS19.0 was used to perform the principal component analysis of health service utilization indicators. Differences were considered statistically significant with a bilateral P<0.05 in all tests.

#### Descriptive analysis

This study quantitatively compared the differences and trends in the utilization of basic medical services between urban and suburban areas using a descriptive statistical analysis of the mean and rate of each basic medical service utilization index. The average annual growth rate (AAGR) was used to compare the range of variation of each basic medical service utilization index. The following formula was used for the calculation:

AAGR=(X2014X20095−1)×100% (1)

where X_2009_ and X_2014_ represent each index from 2009 to 2014, respectively.

#### Comprehensive analysis of basic medical service utilization

Principal component analysis is a statistical method that is applied to evaluate the relationship between each index by dimensionality reduction to convert multiple indicators into a few unrelated indicators, thus simplifying further research[20]. This technique fundamentally solves the problem of information overlap between indicators[21] and greatly simplifies the structure of the original index system.

#### Predicted analysis

The ARIMA prediction model was used to predict data for 2014 (2015) and 2018. The ARIMA (p, d, q) model is known as the autoregressive integrated moving average model, where AR is autoregressive, P is the autoregressive term, MA is the moving average, q is the moving average items, and d is the differential times when the time series is stable[20].

### Quality control

All personnel involved in the field investigation were strictly trained and tested. The questionnaire was standardized and unified to ensure the quality of the survey. During the study design process, domestic experts were invited to guide the development of the questionnaire and the entire survey program. Preliminary tests using the questionnaire presented in Attachment 1 were conducted at one community health service centre. Availability and operability of the data collection were evaluated. According to the preliminary tests, some adjustments were made to the questionnaire in order to make the questionnaire better reflect the real situation of the institutions investigated (e.g., the format of the tables, the logical relationships between each entry, and the units of some survey indicators).

## Results

### Predicted trends and variation of community health service center populations in urban and suburban areas in Shanghai from 2009 to 2013

#### Comparisons of total populations serviced in urban and suburban areas

During the five years, from 2009 to 2013, the total urban population receiving services was larger than in the suburbs (P<0.001). The total population receiving services in urban areas displayed an average annual growth rate of 1.43%, which was smaller than that in the suburbs (4.91%). The total serviced population was estimated to increase to 85,471 by 2018.

#### Comparisons of registered household populations in urban and suburban areas

During the five years, from 2009 to 2013, the absolute number of the total registered household population that received services in urban areas was higher than that in the suburbs (P<0.001). The average annual growth rate in the suburbs (1.27%) was higher than that in urban areas (0.07%). The registered household population was estimated to reach 55,785 by 2018.

#### Comparisons of people aged sixty years and older serviced in urban and suburban areas

During the five-year period, the absolute number of people aged ≥60years who were serviced in urban areas was significantly higher than that in the suburbs (P<0.001). Urban areas had an average annual growth rate of 4.68%, which was lower than that in the suburbs (6.92%). The elderly aged ≥60 years receiving service was predicted to reach 12,788 by 2018.

#### Comparisons of children <6 years of age serviced in urban and suburban areas

During the five-year period, the number of children <6 years of age who were serviced in urban areas was higher than that in the suburbs (P<0.001). In addition, the average annual growth rate of 5.65% in urban areas was higher than that in the suburbs (4.52%). The number of children <6 years of age was predicted to increase to 5,152 by 2018 (Tables 1 and 2).

**Table 1:** Comparisons on service population of community health service centers in urban and suburban areas of Shanghai from 2009 to 2013.

Years	Urban								Suburban								*T*				*P*			
	Total population serviced in the community		Household registered population		People aged 60 and above		Children under age 6		Total population serviced in the community		Household registered population		People aged 60 and above		Children under age 6		Total population serviced in the community	Household registered population	People aged 60 and above	Children under age 6	Total population serviced in the community	Household registered population	People aged 60 and above	Children under age 6
	M	SD	M	SD	M	SD	M	SD	M	SD	M	SD	M	SD	M	SD								
2009	89801.4	38528.3	78783.3	28045.7	17970.9	6369.0	3062.0	1627.6	62526.5	42670.7	33217.6	19607.0	7225.7	3760.2	1876.7	1985.4	2.595	8.166	7.972	2.609	0.012	<0.001	<0.001	0.011
2010	95929.3	35387.2	81025.5	28062.6	18954.9	6777.3	3133.4	1848.6	67271.8	51163.7	33753.0	19931.7	7431.3	4138.0	1973.7	2089.8	2.459	8.047	9.035	2.369	0.017	<0.001	<0.001	0.021
2011	96144.4	34785.8	81081.7	28226.0	19807.6	7349.5	3244.4	1883.5	70959.9	56130.5	34168.2	19780.6	7605.2	4288.4	2101.4	2298.2	2.025	8.345	7.981	2.201	0.047	<0.001	<0.001	0.031
2012	95559.8	38733.4	80001.3	28539.5	20419.5	7798.2	3442.6	2034.5	72432.0	56920.7	34468.8	19950.9	8114.0	4600.0	2183.4	2298.0	1.801	8.019	7.571	2.368	0.076	<0.001	<0.001	0.021
2013	95067.1	42612.9	81011.2	28484.7	21574.8	8421.5	3814.5	2232.7	75737.1	60629.5	34933.1	20376.4	9442.5	7624.5	2240.0	2314.7	1.403	8.048	6.350	2.852	0.165	<0.001	<0.001	0.006
09–13	94538.3	42612.9	80380.6	28484.7	19758.3	8421.5	3342.9	2232.7	69785.4	60659.5	34108.1	19761.0	7963.7	7624.5	2075.0	2314.7	4.967	18.523	-13.163	0.279	<0.001	<0.001	<0.001	<0.001

**Table 2:** Comparisons of AAGR of community health service index in urban and suburban areas of Shanghai from 2009 to 2014 (2013).

Community health service index	Urban Areas	Suburban Areas
Total population serviced in the community	1.43%	4.91%
Household registered population	0.70%	1.27%
People aged sixty and above	4.68%	6.92%
Child under age 6	5.65%	4.52%
Number of annual outpatient visits	4.07%	7.30%
Outpatient expenditure per-time	2.87%	1.21%
Home bed-day costs	10.07%	47.75%
Behavior correction rate of hypertension	3.25%	3.81%
Hypertension control rate	2.05%	2.70%
Behavior correction rate of DM	2.61%	3.63%
DM control rate	4.71%	5.02%
Overall satisfaction	0.45%	1.10%
Case number of referral to superior hospital	4.93%	11.17%
Case number of referral to inferior hospital	-5.48%	18.01%

### Single factor analysis and comparison of the range of variation in community basic medical service quality in urban and suburban areas in Shanghai from 2009 to 2014

#### Comparison of the efficiency of basic medical service provisions in urban and suburban areas

Based on the absolute number of annual outpatient registrations, the annual number of registered outpatients in urban areas (increased from 364,375 to 427,495) was higher than that in the suburbs (increased from 135,244 to 179,275) during the five-year period (P<0.001). The average annual growth rate in urban areas (4.07%) was lower than that in the suburbs (7.30%). The number of annual outpatient visits was expected to increase to 255,700 by 2018 (Tables 2 and 3).

**Table 3:** Comparisons on basic medical service efficiency of community health service centers in urban and suburban areas of Shanghai from 2009 to 2013.

Year	Urban		Suburban		*T*	*P*
	Number of annual outpatient visits		Number of annual outpatient visits		Number of annual outpatient visits	Number of annual outpatient visits
	M	SD	M	SD		
2009	364375	129986.6	135244	110476.3	7.887	<0.001
2010	408816	130649.1	149262	128986.3	8.319	<0.001
2011	411669	122281.6	148224	124970.9	8.830	<0.001
2012	402633	112947.8	162286	142480.3	7.563	<0.001
2013	427495	115157.2	179275	166013.5	6.933	<0.001
2009–2013	364375	129986.5	135244	110476.3	7.887	<0.001

#### Comparison of the benefits of basic medical service provision in urban and suburban areas

Over the six years, the outpatient expenditure per-time was higher in urban areas (from 116 to 134 Yuan) than in the suburbs (from 105 to 111 Yuan) (P<0.001). Urban areas had an average annual growth rate of 2.87%, which was higher than that in the suburbs (1.21%). Outpatient expenditure per-time waspredicted to decrease to 109 Yuan overall in 2018, and in urban and suburban areas it waspredicted to drop to 123 and 105 Yuan.

During the six years, home bed-day costs in urban areas (from 36 to 63 Yuan) were higher than those in the suburbs (from 6 to 51 Yuan) (P = 0.022). Urban areas had an average annual growth rate of 10.07%, which was lower than that in the suburbs (47.75%). Home bed-day costs wereestimated to decrease to 40 Yuan by 2018, corresponding to a rate of 59 Yuan in urban areas and 28 Yuan in the suburbs (Tables 2 and 4).

**Table 4:** Comparisons on health service benefit of community health service centers in urban and suburban areas of Shanghai from 2009 to 2014.

Year	Urban				Suburban				*T*		*P*	
	Outpatient expenditure per-time		Home bed-day costs		Outpatient expenditure per-time		Home bed-day costs		Outpatient expenditure per-time	Home bed-day costs	Outpatient expenditure per-time	Home bed-day costs
	M	SD	M	SD	M	SD	M	SD				
2009	116	12.3	36	65.1	105	20.4	6	7.6	2.528	2.089	0.014	0.049
2010	115	10.4	59	103.9	103	21.4	7	8.1	2.664	2.390	0.010	0.026
2011	113	8.3	62	118.9	99	23.6	9	9.7	3.053	2.138	0.003	0.044
2012	122	11.5	56	96.5	103	25.6	25	77.6	3.769	1.302	<0.001	0.199
2013	125	11.4	57	93.8	109	24.7	40	110.0	3.162	0.608	0.002	0.546
2014	134	13.6	63	98.7	111	26.6	51	143.7	3.897	0.349	<0.001	0.728
2009–2014	121	13.2	36	65.1	105	24.0	6	7.6	8.746	0.022	<0.001	0.022

#### Comparison of management effects on common chronic diseases in urban and suburban areas

During the six years, the rate of hypertension behavior correction in urban areas increased from 63.1% in 2009 to 74.1% in 2014, which was higher than the increase from 60.6 to 72.9% in the suburbs, but this difference was not statistically significant (P = 0.595). The rate of hypertension behavior correction in urban areas had an average annual growth rate of 3.25%, which was lower than that in the suburbs (3.81%). By 2018, the hypertension behavior correction rate was predicted to decrease to 68.6%, corresponding to 70.2% in urban areas and 68.1% in the suburbs.

Over the six years, the hypertension control rate in urban areas increased from 67.8% in 2009 to 75.0% in 2014, which was significantly lower than that in the suburbs (73.4 to 83.9%, P = 0.037). The hypertension control rate in urban areas had an average annual growth rate of 2.05%, which was lower than that in the suburbs (2.70%). The hypertension control rate was estimated to decrease to 77.4% by 2018, corresponding to rates of 74.7% in urban areas and 79.0% in the suburbs (Tables 2 and 5).

**Table 5:** Comparisons on hypertension management effect of community health service centers in urban and suburban areas of Shanghai from 2009 to 2014.

Year	Urban		Suburban		*T*		*P*	
	Behavior correction rate of hypertension	Hypertension control rate	Behavior correction rate of hypertension	Hypertension control rate	Behavior correction rate of hypertension	Hypertension control rate	Behavior correction rate of hypertension	Hypertension control rate
2009	63.1	67.8	60.5	73.4	0.284	-1.303	0.778	0.197
2010	65.1	73.7	63.8	76.3	0.151	-0.644	0.881	0.522
2011	65.9	72.4	64.3	76.0	0.18	-0.842	0.858	0.403
2012	71.3	76.0	67.4	76.6	0.472	-0.142	0.64	0.887
2013	70.8	77.0	70.7	77.6	0.009	-0.139	0.993	0.89
2014	74.1	75.0	72.9	83.9	0.161	-2.544	0.873	0.013
2009–2014	68.4	73.6	66.7	77.3	0.533	-2.095	0.595	0.037

During the six years, the behavior correction rate of DM in urban areas increased from 66.4% in 2009 to 75.5% in 2014, which was significantly higher than that in the suburbs (53.2 to 63.6%, P = 0.001). The behavior correction rate of DM in urban areas had an average annual growth rate of 2.61%, which was lower than that in the suburbs (3.63%). The behavior correction rate of DM was predicted to decrease to 64.4% in 2018, corresponding to 72.1% in urban areas and 60.5% in the suburbs.

During the six years, the control rate of DM in urban areas increased from 61.8% in 2009 to 77.7% in 2014, which was significantly higher than that in the suburbs (53.0 to 59.8%, P<0.001). The control rate of DM in urban areas had an average annual growth rate of 4.71%, which was lower than that in the suburbs (5.02%). The control rate of DM wasestimated to decrease to 65.0% by 2018, corresponding to a decrease to 73.4% in urban areas, whereas that in the suburbs will increase to 73.3% (see Tables 2 and 6).

**Table 6:** Comparisons on DM management effect of community health service centers in urban and suburban areas of Shanghai from 2009 to 2014.

Year	Urban		Suburban		*T*		*P*	
	Behavior correction rate of DM	DM control rate	Behavior correction rate of DM	DM control rate	Behavior correction rate of DM	DM control rate	Behavior correction rate of DM	DM control rate
2009	66.4	61.8	53.2	53.0	1.334	1.445	0.190	0.153
2010	68.3	71.7	56.2	53.1	1.347	3.171	0.186	0.002
2011	70.8	67.7	57.7	55.4	1.502	2.361	0.142	0.021
2012	72.3	70.3	61.1	59.3	1.324	2.341	0.194	0.022
2013	73.3	76.8	64.1	70.4	1.104	1.665	0.277	0.101
2014	75.5	77.7	63.6	67.6	1.434	2.513	0.160	0.014
2009–2014	71.1	71.1	59.4	59.8	3.306	5.654	0.001	<0.001

#### Comparison of the overall satisfaction of community residents in urban and suburban areas

Over the six years, the overall satisfaction of residents in urban areas increased from 94.3% in 2009 to 96.4% in 2014, which was significantly higher than that in the suburbs (86.8 to 91.7%, P<0.001). The overall satisfaction in urban areas had an average annual growth rate of 0.45%, which was lower than that in the suburbs (1.11%). The overall satisfaction of community residents was predicted to increase to 94.8% by 2018, corresponding to rates of 97.2% in urban areas and 93.2% in the suburbs (Tables 2 and 7).

**Table 7:** Comparisons on overall satisfaction of community health service centers residents in urban and suburban areas of Shanghai from 2009 to 2014.

Year	Urban	Suburban	*T*	*P*
2009	94.3	86.8	1.67	0.102
2010	94.9	88.4	1.478	0.146
2011	95.3	89.1	1.394	0.170
2012	95.4	90.1	1.223	0.228
2013	96.1	91.1	1.121	0.268
2014	96.4	91.7	1.075	0.288
2009–2014	95.4	89.5	4.276	<0.001

#### Comparison of the two-way referral effect in urban and suburban areas

During the five years, the referral of patients to a superior hospital in urban areas increased from 1293 referrals in 2009 to 1645 in 2013, which was lower than that in the suburbs (1398 to 2375 referrals), but this difference was not statistically significant (P = 0.161). Case referral to a superior hospital in urban areas had an average annual growth rate of 4.93%, which was lower than that in the suburbs (11.17%). Patient referral to a superior hospital waspredicted to increase to 3240 by 2018, of which 1658 cases will occur in urban areas and 3548 in the suburbs.

During the five years, patient referral to an inferior hospital in urban areas decreased from 167 in 2009 to 126 in 2013. This was significantly lower than that reported in the suburbs, which demonstrated an increase from 305 to 698 (P = 0.001). Case referral to an inferior hospital in urban areas had an average annual growth rate of -5.48%, which was lower than that in the suburbs (18.01%). The number of cases referred to an inferior was estimated to increase to 383 cases by 2018, of which 136 cases will occur in urban areas and 559 in the suburbs (Tables 2 and 8).

**Table 8:** Comparisons on interactions amount between community health service centers and superior hospitals in urban and suburban areas of Shanghai from 2009 to 2013.

Years	Urban				Suburban				*T*		*P*	
	Case number of referral to superior hospital		Case number of referral to inferior hospital		Case number of referral to superior hospital		Case number of referral to inferior hospital		Case number of referral to superior hospital	Case number of referral to inferior hospital	Case number of referral to superior hospital	Case number of referral to inferior hospital
	M	SD	M	SD	M	SD	M	SD				
2009	1293	607.4	167	300.6	1398	2322.0	305	658.5	-0.258	-0.879	0.797	0.376
2010	1523	775.6	158	333.6	1511	2574.9	304	726.7	0.028	-0.887	0.977	0.381
2011	1576	798.3	129	262.3	1789	2682.1	531	1305.4	-0.461	-1.528	0.647	0.138
2013	1690	853.2	123	218.8	2011	2638.2	899	2040.7	-0.708	-1.924	0.483	0.065
2013	1645	782.8	126	197.6	2375	2286.9	598	1544.1	-1.843	-1.938	0.071	0.063
09–13	1553	771.9	140	260.8	1827	2505.5	555	1370.7	-1.406	-3.360	0.161	0.001

### Principal component analysis of basic medical service utilization in urban and suburban areas

A principal component analysis was conducted by analyzing21 basic medical service utilization indicators, and the comprehensive evaluation of health service utilization was finally completed.

#### KMO and Bartlett’s spherical degree test

In this study, the appropriate detection value for the KMO sample was 0.5, which demonstrated that the partial correlation between the test variables was small and that the data could be used for factor analysis. The chi-square value of Bartlett’s spherical degree test was 1068.054, and the degree of freedom was 210 (P<0.001), which would be considered a non-unit matrix. The factor model was therefore suitable.

#### Characteristic value and contribution rate of the variance

The first seven principal components were obtained using the variable characteristic value (λ≥1) as the standard. Their variances were 4.917, 3.317, 2.647, 1.901, 1.303, and 1.058, respectively, and their cumulative contribution rate to the variance reached 82.138%, which could better represent the information obtained for the original 21 indicators (show Table 9).

**Table 9:** Total variance explained.

Principal component	Initial eigenvalue			Extraction sums of squared loadings		
	Total	% of Var	Cumulative %	Total	% of Var	Cumulative %
1	4.917	23.414	23.414	4.917	23.414	23.414
2	3.317	15.797	39.211	3.317	15.797	39.211
3	2.647	12.603	51.814	2.647	12.603	51.814
4	2.116	10.075	61.889	2.116	10.075	61.889
5	1.901	9.051	70.940	1.901	9.051	70.940
6	1.303	6.207	77.147	1.303	6.207	77.147
7	1.058	5.036	82.183	1.058	5.036	82.183

#### Factor loading matrix before rotation

The factor loading matrix before rotation (a_ij_) consisted of 21 basic medical service utilization variables and the extraction of the seven principal components related to the load vector matrix; that served as the correlation coefficient between the 21 variables and the principal component, which was the basis for calculating the normalized orthogonal eigenvectors.

#### Standard orthogonal feature vector matrix

The standard orthogonal feature vector matrix (e_ij_) was calculated according to the formula

eij=aijλi, (2)

where a_ij_ is the principal component load vector and $\sqrt{\lambda_{i}}$ is the arithmetic square root of each principal component characteristic value.

#### Principal component scores and comprehensive score

The principal component scores were calculated according to the formula

$$y=Z_{x}\times e_{ij}$$ (3),

where Z_x_ is the matrix after the 21 variables were standardized, and e_ij_ is the normalized orthogonal feature vector matrix. Finally, the comprehensive score was calculated according to the variance contribution rate and scores for the principal component *y*_*CS*_ = 0.23414*y*_1_ + 0.15797*y*_2_ + 0.12603*y*_3_ + 0.10075*y*_4_ + 0.09051*y*_5_ + 0.06207*y*_6_ + 0.05036*y*_7_ (In this study, 21 basic medical service utilization variables were introduced: the first principal component score above 0.3 was the hypertension behavior correction rate, the diabetes mellitus behavior correction rate, and tumor awareness; the second was the hypertension management rate; the third was the upward-referral cases and hospital bed-day costs; the fourth was outpatient expenditure per-time; the fifth was the diabetes mellitus management rate; the sixth was the downward-referral cases; and the seventh was the total outpatient visits. A comprehensive score above 0.8 represented the behavior correction rate for hypertension, diabetes mellitus, and tumor awareness and control rate; the other details are listed in Table 10.

**Table 10:** Component and comprehensive score.

#	Variable	Component 1	Component 2	Component 3	Component 4	Component 5	Component 6	Component 7	Total
1	Behavior correction rate of hypertention	.383	.042	.001	.003	.010	.009	.001	.098
2	Behavior correction rate of DM	.365	.041	.000	.026	.000	.011	.002	.095
3	Tumor awareness rate	.360	.001	.005	.001	.033	.002	.020	.089
4	Behavior correction rate of tumor	.217	.224	.000	.008	.000	.000	.000	.087
5	Tumor control rate	.159	.217	.033	.007	.021	.009	.016	.080
6	DM management rate	.140	.056	.001	.032	.311	.024	.000	.075
7	DM awareness rate	.137	.121	.000	.034	.138	.058	.003	.071
8	Case number of referral to inferior hospital	.116	.051	.069	.005	.003	.0328	.007	.065
9	Hypertension awareness rate	.100	.110	.000	.092	.186	.050	.003	.070
10	DM control rate	.056	.019	.019	.210	.110	.026	.076	.055
11	Tumor management rate	.055	.032	.142	.031	.085	.098	.108	.058
12	Total number of hospital bed-days	.036	.106	.242	.132	.016	.001	.008	.071
13	Case number of referral to superior hospital	.034	.015	.352	.000	.000	.249	.003	.070
14	Hypertension control rate	.018	.001	.132	.000	.120	.040	.233	.046
15	Hospital bed-days costs	.016	.031	.308	.002	.007	.027	.121	.055
16	Hypertension management rate	.009	.314	.007	.098	.042	0.18	.000	.067
17	Number of annual patient visits	.006	.225	.079	.201	.011	.001	.000	.068
18	Home bed-day costs	.006	.025	.098	.002	.040	.113	.056	.032
19	Overall satisfaction	.005	.003	.190	.057	.236	.003	.017	.054
20	Outpatient expenditure per time	.001	.087	.002	.91	.000	.059	.003	.057
21	Number of total out-call visits	.000	.102	.007	.118	.007	.015	.348	.048

#### Comparison of the principal component scores and comprehensive scores for urban and suburban areas

**Fig 1:**
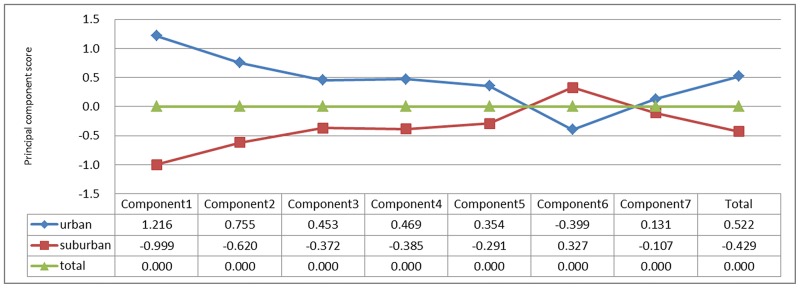
Principal component analysis of basic medical service utilization of community health service centers in urban and suburban areas of Shanghai from 2009 to 2014.

The urban areas had six principal component scores that were superior to those in the suburbs for the seven proposed principal components, and there were significant differences among principal components 1, 2, 4 and 6 (P = <0.001, 0.004, 0.036, and 0.022, respectively). The comprehensive score for the urban areas was superior to that for the suburbs (P<0.001) (Fig 1).

## Discussion

This cross-sectional study compared the utilization of community basic medical services in Shanghai urban and suburban areas between 2009 and 2014. During the 6-year study period, there was an increasing trend toward greater efficiency of basic medical service provision, benefits of basic medical service provision, effectiveness of common chronic disease management, overall satisfaction of community residents, and two-way referral effects. In addition to the implementation effect of hypertension management and two-way referral, the remaining indicators showed a superior effect in urban areas compared with the suburbs. In addition, among the seven principal components, four principal component scores were better in urban areas than in suburban areas. The urban comprehensive score also exceeded that of the suburbs.

The key point to the development of medical and health care is not to provide clinical medical services but to provide preventive health care services. A large number of common and frequently occurring diseases can be adequately treated at basic health institutions. Community health service plays an effective role in achieving health care equity and efficiency, controlling the growth of medical expenditure and improving the health level of community residents. Nevertheless, foreign medical practice has shown that there is an imbalance in the quality of urban and suburban community health services[4, 5, 22, 23]. In this article, we discussed the comparative results of the health service utilization quality of urban and suburban community health service centers in Shanghai to address their effectiveness and developing health service trends.

### Important conclusions of the study

The results suggested that the absolute numbers of the total serviced population, household registered population, population ≥60 years of age, and population of children <6 years in urban areas were higher than that in the suburbs between 2009 and 2013 (P<0.001). The average annual growth rate of the registered household population and children <6 years of age was greater in urban areas than in the suburbs. The total serviced population, household registered population, and population of children <6 years were predicted to show increasing trends by 2018, which is consistent with the findings of Zhang An and Bao Yong [24]. These results suggest that although China has invested a large amount of manpower and material and financial resources in the implementation of an affordable medical service system, this goal has not been completely reached [25]. In addition, health service demands continue to grow, particularly in an international metropolis such as Shanghai where they will continue to grow over the next five years, with greater needs in urban areas than in suburban areas. Under the premise of limited resources, research and practice with the goal of improving the efficiency of health care system utilization is very important [26].

From 2009 to 2014 (partial data for 5 years), the efficiency of basic medical service provision indicators has shown a greater rate of annual outpatient visits in urban areas than in the suburbs (P<0.001), whereas the average annual growth rate was lower in urban areas than in the suburbs. Annual outpatient visits are estimated to show a growing trend by 2018. The benefits of basic medical service provision indicators showed that outpatient expenditure per-time and home bed-day costs were higher in urban areas than in the suburbs (P<0.001 and P = 0.022, respectively), and the average annual growth rate of annual outpatient visits in urban areas was higher than that in the suburbs. By contrast, the growth rate of home bed-day costs was lower in urban areas than in the suburbs. Common chronic disease management effect indicators showed that with the exception of the hypertension control rate in urban areas, which was lower than that in the suburbs (P = 0.037), the behavior correction rate and control rate of diabetes mellitus was higher in urban areas than in the suburbs (P = 0.001 and P<0.001, respectively). In addition, the average annual growth rate of the behavior correction rate and the control rate of hypertension and diabetes mellitus in urban areas were lower than those in the suburbs. Research by Sun Xiaoming [27]further suggested that almost all investigated communities have shown some improvement in performance over time, but we must recognize that health care reform in rural areas is a gradual process, and it takes time to exert a profound impact. Differences in the performance of the community health service centers can be attributed to social characteristics and the implementation of health care policies. The best performance score is often concentrated in rural demonstration centers with high-density population, but the performance of rural or suburban areas was ranked lower, and they appeared to have a weak capacity to provide adequate health care services and an improved working environment [28].

During the six-year study period, the survey results showed an increasing trend in satisfaction, regardless of whether they were urban or suburban community residents, but the overall satisfaction of urban residents was higher than that in the suburbs (P<0.001), whereas its average annual growth rate was lower than that in the suburbs. An increasing trend in overall resident satisfaction was predicted by 2018, in agreement with the conclusions of Sun Xiaoming[27]fromFudan University. The comprehensive satisfaction of the investigated community health centers was significantly different during the three years. Moreover, another study in China showed that outpatient and inpatient satisfaction in China was associated with the type of health care service. The satisfaction of patients seeking outpatient services from a community health center was high. Patients at county and tertiary hospitals consistently complained about the waiting time, bad attitude of health personnel, and high costs of treatment, and the overall satisfaction of outpatients was lower[29]. Regarding patient satisfaction, community health centers and hospitals face different challenges. To improve patient satisfaction, further health care reform is necessary in China (e.g., improving the quality of primary health care, establishing a referral system, among others)[29].

China is making efforts to build a community primary diagnosis, medical grade, acute and chronic treatment, two-way referral treatment model. Many residents consider the quality of service provided by community health service centers to be insufficient [30]. Consequently, the excessive use of large hospitals and inadequate use of primary health care services by patients is a widespread problem in China[30]. Community first diagnosis and two-way referrals between community and general hospitals were the weak links in the utilization of community health service in Kunming [11]. The present study also suggested that although the total number of two-way referrals increased annually during the five years of study, the suburbs more effectively implemented two-way referrals than did urban areas (P<0.001). This finding showed that the two-way referral function of community health service utilization in urban areas of Shanghai must be further strengthened.

In the chapter evaluating the performance of the community health service center in Pudong New Area, Shanghai, Sun Xiaoming [27] at Fudan University highlighted that the overall performance of the investigated community health service centers increased annually over the past three years. Basic medical services, public health services and comprehensive satisfaction were significantly different. Similar conclusions were drawn in the present study; during the 6 years (a portion for 5 years), urban areas were superior to the suburbs in terms of the efficiency of basic medical service provision (number of annual outpatient visits), benefits of basic medical service provision (outpatient expenditure per-time, hospital bed-day costs), common chronic disease management effects (behavior correction rate and control rate of diabetes mellitus), and overall satisfaction of community residents. In a comprehensive evaluation of basic medical service utilization, the same conclusion was reached by Ma Yanan[8]. Seven principal components were extracted, and six principal component scores were superior in urban areas compared to suburban areas (principal components 1, 2, 4, and 6, P = <0.001, 0.004, 0.036, and 0.022, respectively). The comprehensive score was also better in urban areas than in suburban areas (P<0.001).

### Contribution of the research in China

First, based on routinely collected data by a community health service center, the research focused on analyzing basic medical service utilization quality in urban and suburban areas of Shanghai. Over 6 years, a large sample size with continuity and comparability from 73 community health service centers was investigated, which compensates for the shortage of research on health service utilization quality using mostly cross-sectional survey data in China and abroad. Second, applying principal component analysis can fundamentally solve the problem of information overlap among indicators [21] and greatly simplify the structure of the original index system. The weight of the integrated factors in the principal component analysis is not determined artificially, but according to the contribution rate of the comprehensive factors [31]. Thus, principle component analysis overcomes the limitations of arbitrarily determined weights in some evaluation methods, making the results of the comprehensive evaluation unique, objective and reasonable [8]. Third, indicators that showed changes in the range and time prediction method were used to circumvent the problem of a single application method in the research, and this was the focus of the study. In addition, this study also considered the community health center as the research subject; thus, the availability and reliability of the data were superior to those when considering community residents as the subject. In addition, most domestic scholars have focused on the study of urban and rural community health services, but research investigating urban and suburban community health services is quite rare. Therefore, the results of this study could provide a basis for strategies to improve the quality of basic medical services in the investigated localities, simultaneously providing a reference value for the development and planning of community health in other areas of the country.

### Limitations of the research

Notably, we only analyzed the quality status of basic medical service utilization in urban and suburban areas according to the variables in the community health service center database, and further discussion of other factors beyond this database that may affect the quality of basic medical service utilization, such as the reactivity of non-medical services, should be further considered [32]. Because of space limitations, data and discussion related to health resource allocation and public health service utilization in this study will be submitted in a separate manuscript. In the discussion of resource allocation, the relationship between community health service quantity and the number of practicing physicians in urban and suburban areas, accounting for the number of practicing physicians and diagnosis and the treatment visits, will notbe mentioned in this article.

### Conclusion

In summary, our study showed that, over the 6 years of study, basic medical service utilization has maintained a rapid increasing trend in Shanghai, and comprehensive satisfaction has clearly improved, but there is an imbalance in health service utilization between urban and suburban areas. The health administrative department should solve the imbalance between urban and suburban institutions and provide the necessary supports to the underdeveloped areas. For optimal results, the health care system should focus on referral criteria and on improving communication between primary care physicians and residents [33]. Further measures should be focused on health care reform to improve patient satisfaction, for example, by improving the quality of primary health care and setting up a referral medical system, among others [29].

## Supporting information

S1 Table The questionnaire used to survey the hospitals, which contains seven parts: Service population, revenue and expenditure, human resources, services, overall resident satisfaction, two-way referral, and information. (DOCX)

S1 Data The raw data from the questionnaires. (XLS)
