# Supplementary material for: Quality of community basic medical service utilization in urban and suburban areas in Shanghai from 2009 to 2014
Source: PLoS One. 2018 May 23;13(5):e0195987. doi: 10.1371/journal.pone.0195987 (PMC5965823; doi:10.1371/journal.pone.0195987)
Supplement: S1 Table — (DOCX) [file pone.0195987.s001.docx]

**Attachment 1**

**Questionnaire on Deepening Reform of Community Health Service in Shanghai (English version)**

**First Part Service population situation (unit: People)**

| Total population serviced in the community | 2009 | 2010 | 2011 | 2012 | 2013 |
| --- | --- | --- | --- | --- | --- |
| 1 Household registered population |  |  |  |  |  |
| 1.1 There into: the male sex |  |  |  |  |  |
| 1.2 the female sex |  |  |  |  |  |
| 1.3 People aged sixty and above |  |  |  |  |  |
| 1.4 Child under age six |  |  |  |  |  |

**Second Part Revenue and Expenditure Situation**

1 Center income (unit: ten thousand, keep two decimals)

|  | 2009 | 2010 | 2011 | 2012 | 2013 |
| --- | --- | --- | --- | --- | --- |
| Total income |  |  |  |  |  |
| 1 Financial allocation |  |  |  |  |  |
| 1.1 Special funds |  |  |  |  |  |
| 1.2 Funds for prevention and protection |  |  |  |  |  |
| 2 Superior subsidy |  |  |  |  |  |
| 3 Medical income |  |  |  |  |  |
| 4 Drug revenue |  |  |  |  |  |
| 4.1 Traditional Chinese medicine income |  |  |  |  |  |
| 5 Other income |  |  |  |  |  |

2 Medical Insurance (unit: ten thousand yuan, keep two decimals)

|  | 2009 | 2010 | 2011 | 2012 | 2013 |
| --- | --- | --- | --- | --- | --- |
| Medical insurance prepayment |  |  |  |  |  |

3 Central Expenditure Situation (unit: ten thousand yuan, keep two decimals)

|  | 2009 | 2010 | 2011 | 2012 | 2013 |
| --- | --- | --- | --- | --- | --- |
| Total expenditure |  |  |  |  |  |
| 1 Salary and welfare |  |  |  |  |  |
| 2 Commodity and service expenditure |  |  |  |  |  |
| 2.1 Drug cost |  |  |  |  |  |
| 3 Personal and family subsidy expenditure |  |  |  |  |  |
| 4 Other capital expenditure |  |  |  |  |  |

4 Balance of Payments (unit: ten thousand yuan, keep two decimals)

|  | 2009 | 2010 | 2011 | 2012 | 2013 |
| --- | --- | --- | --- | --- | --- |
| Balance of payments |  |  |  |  |  |

**Third Part Personnel Situation**

1 General Situation of Staff (unit: people)

|  | 2009 | | 2010 | | 2011 | | 2012 | | 2013 | |
| --- | --- | --- | --- | --- | --- | --- | --- | --- | --- | --- |
|  | permanent staff | temporary staff | permanent staff | temporary staff | permanent staff | temporary staff | permanent staff | temporary staff | permanent staff | temporary staff |
| Total staff |  |  |  |  |  |  |  |  |  |  |
| Clinical doctor |  |  |  |  |  |  |  |  |  |  |
| Public health staff |  |  |  |  |  |  |  |  |  |  |
| Nursing staff |  |  |  |  |  |  |  |  |  |  |
| Pharmacy staff |  |  |  |  |  |  |  |  |  |  |
| Laboratory staff |  |  |  |  |  |  |  |  |  |  |
| Medical images staff |  |  |  |  |  |  |  |  |  |  |
| Other health technicians |  |  |  |  |  |  |  |  |  |  |
| Administrative personnel |  |  |  |  |  |  |  |  |  |  |
| Logistic personnel |  |  |  |  |  |  |  |  |  |  |

Note: According to the post classification, such as doctors and nurses engaged in public health work should be all included in the public health workers.

2 Staff Qualifications Condition (unit: people)

|  | 2009 | | 2010 | | 2011 | | 2012 | | 2013 | |
| --- | --- | --- | --- | --- | --- | --- | --- | --- | --- | --- |
|  | permanent staff | temporary staff | permanent staff | temporary staff | permanent staff | temporary staff | permanent staff | temporary staff | permanent staff | temporary staff |
| Master |  |  |  |  |  |  |  |  |  |  |
| Undergraduate |  |  |  |  |  |  |  |  |  |  |
| Junior college |  |  |  |  |  |  |  |  |  |  |
| Technical secondary school |  |  |  |  |  |  |  |  |  |  |
| Non-educational background personnel |  |  |  |  |  |  |  |  |  |  |
| Total |  |  |  |  |  |  |  |  |  |  |

|  | 2009 | | 2010 | | 2011 | | 2012 | | 2013 | |
| --- | --- | --- | --- | --- | --- | --- | --- | --- | --- | --- |
|  | permanent staff | temporary staff | permanent staff | temporary staff | permanent staff | temporary staff | permanent staff | temporary staff | permanent staff | temporary staff |
| High level |  |  |  |  |  |  |  |  |  |  |
| Intermediate level |  |  |  |  |  |  |  |  |  |  |
| Primary level |  |  |  |  |  |  |  |  |  |  |
| Probation period |  |  |  |  |  |  |  |  |  |  |
| Personnel without professional title |  |  |  |  |  |  |  |  |  |  |
| Total |  |  |  |  |  |  |  |  |  |  |

3 Professional Title Condition of the Staff (unit: people)

4 Training Situation (unit: people)

|  |  | 2009 | 2010 | 2011 | 2012 | 2013 |
| --- | --- | --- | --- | --- | --- | --- |
| General practitioner | National qualification certificate |  |  |  |  |  |
|  | City-level training certificate |  |  |  |  |  |
|  | Total |  |  |  |  |  |
| Community nurse | National qualification certificate |  |  |  |  |  |
|  | City-level training certificate |  |  |  |  |  |
|  | Total |  |  |  |  |  |
| National qualification  (ministry of human resources and society security issued) | Health management division |  |  |  |  |  |
|  | Dietitian |  |  |  |  |  |
|  | Psychological consultation teacher |  |  |  |  |  |

Note: Obtained national qualification certificate and city-level training certificate at the same time, please fill in the highest level.

**Fourth Part Service situation**

1 Basic Medical Service Efficiency

|  | 2009 | 2010 | 2011 | 2012 | 2013 |
| --- | --- | --- | --- | --- | --- |
| Number of annual outpatient visits |  |  |  |  |  |
| Total number of hospital bed-days |  |  |  |  |  |
| Number of total out-call visits (both medical and nursing) |  |  |  |  |  |

2 Public Health Service Efficiency

2.1 Community Prevention (keep two decimals)

|  | | 2009 | 2010 | 2011 | 2012 | 2013 |
| --- | --- | --- | --- | --- | --- | --- |
| Total incidence rate of infectious diseases (/10^5^) | |  |  |  |  |  |
| Planned immunization coverage rate (%) (based on the same year) | permanent residents |  |  |  |  |  |
|  | floating population |  |  |  |  |  |

2.2 Community Rehabilitation (percentage, keep two decimals)

|  | 2009 | 2010 | 2011 | 2012 | 2013 | 2014 |
| --- | --- | --- | --- | --- | --- | --- |
| Registration rate of mental patients |  |  |  |  |  |  |
| Management rate of mental patients |  |  |  |  |  |  |
| Registration rate of the disabled |  |  |  |  |  |  |
| Management rate of the disabled |  |  |  |  |  |  |

2.3 Community Health Care (percentage, keep two decimals)

|  | 2009 | 2010 | 2011 | 2012 | 2013 | 2014 |
| --- | --- | --- | --- | --- | --- | --- |
| Maternal systematic management rate |  |  |  |  |  |  |
| Systematic management rate of child under age 6 |  |  |  |  |  |  |
| Eye health care rate of people aged 70 and above |  |  |  |  |  |  |
| Health management rate of special population |  |  |  |  |  |  |
| Physical examination rate of kindergartens and students in school |  |  |  |  |  |  |
| Physical examination rate of nursing home and day-care institutions |  |  |  |  |  |  |

Note: Special population includes centenarians, retired cadres, families of revolutionary martyrs and servicemen and so on.

2.4 Community Health Education

|  | 2009 | 2010 | 2011 | 2012 | 2013 |
| --- | --- | --- | --- | --- | --- |
| Organize propaganda column (quantity) |  |  |  |  |  |
| Free distribution of propaganda materials (quantity) |  |  |  |  |  |
| Regular health education lectures (times) |  |  |  |  |  |
| Health consultation (times) |  |  |  |  |  |
| Play recording (times) |  |  |  |  |  |
| Health education prescription (sheets) |  |  |  |  |  |

2.5 Health Index (keep two decimals)

|  | 2009 | 2010 | 2011 | 2012 | 2013 | 2014 |
| --- | --- | --- | --- | --- | --- | --- |
| Infant mortality rate (/10^3^) |  |  |  |  |  |  |
| Child mortality rate under age 5 (/10^3^) |  |  |  |  |  |  |
| Maternal mortality rate (/10^5^) |  |  |  |  |  |  |
| Average life expectancy (year) |  |  |  |  |  |  |

3. Chronic Disease Service Effectiveness (percentage, keep two decimals)

| Disease Category | Effect | 2009 | 2010 | 2011 | 2012 | 2013 | 2014 |
| --- | --- | --- | --- | --- | --- | --- | --- |
| Hypertension | Awareness rate |  |  |  |  |  |  |
|  | Management rate |  |  |  |  |  |  |
|  | Behavior correction rate |  |  |  |  |  |  |
|  | Control rate |  |  |  |  |  |  |
| Diabetes  Mellitus | Awareness rate |  |  |  |  |  |  |
|  | Management rate |  |  |  |  |  |  |
|  | Behavior correction rate |  |  |  |  |  |  |
|  | Control rate |  |  |  |  |  |  |
| Tumor | Awareness rate |  |  |  |  |  |  |
|  | Management rate |  |  |  |  |  |  |
|  | Behavior correction rate |  |  |  |  |  |  |
|  | Control rate |  |  |  |  |  |  |

4 Benefit (unit: yuan, keep one decimals)

|  | 2009 | 2010 | 2011 | 2012 | 2013 | 2014 |
| --- | --- | --- | --- | --- | --- | --- |
| Outpatient expenditure per-time |  |  |  |  |  |  |
| Hospital bed-day costs |  |  |  |  |  |  |
| Home bed-day costs |  |  |  |  |  |  |

**Fifth Part**  **Overall satisfaction of Residents** (percentage, keep two decimal places)

|  | 2009 | 2010 | 2011 | 2012 | 2013 | 2014 |
| --- | --- | --- | --- | --- | --- | --- |
| Overall Satisfaction |  |  |  |  |  |  |

**Sixth Part Two-way Referral (times)**

|  | 2009 | 2010 | 2011 | 2012 | 2013 |
| --- | --- | --- | --- | --- | --- |
| Case number of referral to superior hospital |  |  |  |  |  |
| Case number of referral to inferior hospital |  |  |  |  |  |

**Seventh Part Information Construction**

|  | 2009 | 2010 | 2011 | 2012 | 2013 | 2014 |
| --- | --- | --- | --- | --- | --- | --- |
| Established rate of electronic health care record (yes or no) |  |  |  |  |  |  |
| Information integration of center internal (yes or no) |  |  |  |  |  |  |
| Integration with community health service station (yes or no) |  |  |  |  |  |  |
| Hospital-center-station integration (yes or no) |  |  |  |  |  |  |

**Questionnaire on Deepening Reform of Community Health Service in Shanghai (original Chinese version)**

**上海市社区卫生服务深化改革调查问卷**

一、服务人口情况 (单位：人)

|  | 2009年 | 2010年 | 2011年 | 2012年 | 2013年 |
| --- | --- | --- | --- | --- | --- |
| 所辖社区内的服务总人口数 |  |  |  |  |  |
| 1.户籍人口 |  |  |  |  |  |
| 1.1其中：男性 |  |  |  |  |  |
| 1.2 女性 |  |  |  |  |  |
| 1.3 六十岁以上人口 |  |  |  |  |  |
| 1.4 (0-6岁) 儿童 |  |  |  |  |  |

二、收支情况

1.中心收入情况 (单位：万元，保留两位小数)

|  | 2009年 | 2010年 | 2011年 | 2012年 | 2013年 |
| --- | --- | --- | --- | --- | --- |
| 总收入 |  |  |  |  |  |
| 1财政拨款 |  |  |  |  |  |
| 1.1专项经费 |  |  |  |  |  |
| 1.2防保经费 |  |  |  |  |  |
| 2上级补助 |  |  |  |  |  |
| 3医疗收入 |  |  |  |  |  |
| 4药品收入 |  |  |  |  |  |
| 4.1中药收入 |  |  |  |  |  |
| 5其它收入 |  |  |  |  |  |

2．医保预付情况 (单位：万元，保留两位小数)

|  | 2009年 | 2010年 | 2011年 | 2012年 | 2013年 |
| --- | --- | --- | --- | --- | --- |
| 医保预付金额 |  |  |  |  |  |

3.中心支出情况 (单位：万元，保留两位小数)

|  | 2009年 | 2010年 | 2011年 | 2012年 | 2013年 |
| --- | --- | --- | --- | --- | --- |
| 总支出 |  |  |  |  |  |
| 1．工资福利 |  |  |  |  |  |
| 2．商品和服务支出 |  |  |  |  |  |
| 2.1药品成本 |  |  |  |  |  |
| 3．对个人家庭补助支出 |  |  |  |  |  |
| 4．其他资本支出 |  |  |  |  |  |

4．收支结余情况 (单位：万元，保留两位小数)

|  | 2009年 | 2010年 | 2011年 | 2012年 | 2013年 |
| --- | --- | --- | --- | --- | --- |
| 收支结余情况 |  |  |  |  |  |

三、人力资源情况

1.职工一般情况 (单位：人)

|  | 2009年 | | 2010年 | | 2011年 | | 2012年 | | 2013年 | |
| --- | --- | --- | --- | --- | --- | --- | --- | --- | --- | --- |
|  | 在编 | 非在编 | 在编 | 非在编 | 在编 | 非在编 | 在编 | 非在编 | 在编 | 非在编 |
| 职工总数 |  |  |  |  |  |  |  |  |  |  |
| 临床医生 |  |  |  |  |  |  |  |  |  |  |
| 公卫人员 |  |  |  |  |  |  |  |  |  |  |
| 护理人员 |  |  |  |  |  |  |  |  |  |  |
| 药剂人员 |  |  |  |  |  |  |  |  |  |  |
| 检验人员 |  |  |  |  |  |  |  |  |  |  |
| 影像人员 |  |  |  |  |  |  |  |  |  |  |
| 其他卫技人员 |  |  |  |  |  |  |  |  |  |  |
| 行政人员 |  |  |  |  |  |  |  |  |  |  |
| 后勤人员 |  |  |  |  |  |  |  |  |  |  |

注：按岗位分类。如从事公卫工作的医生和护士都应算入公卫人员内。

2.职工学历情况 (单位：人)

|  | 2009年 | | 2010年 | | 2011年 | | 2012年 | | 2013年 | |
| --- | --- | --- | --- | --- | --- | --- | --- | --- | --- | --- |
|  | 在编 | 非在编 | 在编 | 非在编 | 在编 | 非在编 | 在编 | 非在编 | 在编 | 非在编 |
| 硕士 |  |  |  |  |  |  |  |  |  |  |
| 本科 |  |  |  |  |  |  |  |  |  |  |
| 专科 |  |  |  |  |  |  |  |  |  |  |
| 中专 |  |  |  |  |  |  |  |  |  |  |
| 无学历 |  |  |  |  |  |  |  |  |  |  |
| 合计 |  |  |  |  |  |  |  |  |  |  |

3．职工职称情况 (单位：人)

|  | 2009年 | | 2010年 | | 2011年 | | 2012年 | | 2013年 | |
| --- | --- | --- | --- | --- | --- | --- | --- | --- | --- | --- |
|  | 在编 | 非在编 | 在编 | 非在编 | 在编 | 非在编 | 在编 | 非在编 | 在编 | 非在编 |
| 高级 |  |  |  |  |  |  |  |  |  |  |
| 中级 |  |  |  |  |  |  |  |  |  |  |
| 初级 |  |  |  |  |  |  |  |  |  |  |
| 见习期 |  |  |  |  |  |  |  |  |  |  |
| 无职称 |  |  |  |  |  |  |  |  |  |  |
| 合计 |  |  |  |  |  |  |  |  |  |  |

4．累计培训情况 (单位：人)

|  |  | 2009年 | 2010年 | 2011年 | 2012年 | 2013年 |
| --- | --- | --- | --- | --- | --- | --- |
| 全科医生 | 国家级资质证书 |  |  |  |  |  |
|  | 市级培训证书 |  |  |  |  |  |
|  | 合计 |  |  |  |  |  |
| 社区护士 | 国家级资质证书 |  |  |  |  |  |
|  | 市级培训证书 |  |  |  |  |  |
|  | 合计 |  |  |  |  |  |
| 国家级资质 (人社部办法) | 健康管理师 |  |  |  |  |  |
|  | 营养师 |  |  |  |  |  |
|  | 心理咨询师 |  |  |  |  |  |

注：同时获得国家级资质证书和市级培训证书，填写最高级别。

四、服务情况

1 基本医疗服务效率

|  | 2009年 | 2010年 | 2011年 | 2012年 | 2013年 |
| --- | --- | --- | --- | --- | --- |
| 年门诊挂号人次数 |  |  |  |  |  |
| 住院总床日数 |  |  |  |  |  |
| 总出诊人次数 (包括医疗和护理) |  |  |  |  |  |

2．公共卫生服务效率

2.1社区预防 (保留两位小数)

|  | | 2009年 | 2010年 | 2011年 | 2012年 | 2013年 |
| --- | --- | --- | --- | --- | --- | --- |
| 传染病总发病率 (/十万) | |  |  |  |  |  |
| 规划免疫覆盖率 (%) (以当年覆盖苗种为准) | 常住人口 |  |  |  |  |  |
|  | 流动人口 |  |  |  |  |  |

2.2社区康复 (%，保留两位小数)

|  | 2009年 | 2010年 | 2011年 | 2012年 | 2013年 | 2014年 |
| --- | --- | --- | --- | --- | --- | --- |
| 精神疾病病人建档率 |  |  |  |  |  |  |
| 精神疾病病人管理率 |  |  |  |  |  |  |
| 残疾人建档率 |  |  |  |  |  |  |
| 残疾人管理率 |  |  |  |  |  |  |

2.3 社区保健 (%，保留两位小数)

|  | 2009年 | 2010年 | 2011年 | 2012年 | 2013年 | 2014年 |
| --- | --- | --- | --- | --- | --- | --- |
| 孕产妇系统管理率 |  |  |  |  |  |  |
| 6岁以下儿童系统管理率 |  |  |  |  |  |  |
| 70岁以上老人眼保健率 |  |  |  |  |  |  |
| 特殊人群健康管理率 |  |  |  |  |  |  |
| 幼托机构和在校学生健康体检率 |  |  |  |  |  |  |
| 养老院和日托机构健康体检率 |  |  |  |  |  |  |

注：特殊人群包括百岁老人、离休干部、军烈属等.

2.4 社区健康教育

|  | 2009年 | 2010年 | 2011年 | 2012年 | 2013年 |
| --- | --- | --- | --- | --- | --- |
| 建立宣传栏(期数) |  |  |  |  |  |
| 宣传品免费发放 (数量) |  |  |  |  |  |
| 定期健康教育讲座 |  |  |  |  |  |
| 开展健康咨询 (次数) |  |  |  |  |  |
| 播放录像 (次数) |  |  |  |  |  |
| 健康教育处方 (张数) |  |  |  |  |  |

2.5健康指数 (保留两位小数)

|  | 2009年 | 2010年 | 2011年 | 2012年 | 2013年 | 2014年 |
| --- | --- | --- | --- | --- | --- | --- |
| 婴儿死亡率(/千) |  |  |  |  |  |  |
| 5岁以下儿童死亡率(/千) |  |  |  |  |  |  |
| 孕产妇死亡率(/十万) |  |  |  |  |  |  |
| 平均期望寿命 (岁) |  |  |  |  |  |  |

3. 慢性病服务效果 (%，保留两位小数)

| 病种 | 效果 | 2009年 | 2010年 | 2011年 | 2012年 | 2013年 | 2014年 |
| --- | --- | --- | --- | --- | --- | --- | --- |
| 高血压 | 知晓率 |  |  |  |  |  |  |
|  | 管理率 |  |  |  |  |  |  |
|  | 行为矫正率 |  |  |  |  |  |  |
|  | 控制率 |  |  |  |  |  |  |
| 糖尿病 | 知晓率 |  |  |  |  |  |  |
|  | 管理率 |  |  |  |  |  |  |
|  | 行为矫正率 |  |  |  |  |  |  |
|  | 控制率 |  |  |  |  |  |  |
| 肿瘤 | 知晓率 |  |  |  |  |  |  |
|  | 管理率 |  |  |  |  |  |  |
|  | 行为矫正率 |  |  |  |  |  |  |
|  | 控制率 |  |  |  |  |  |  |

4. 服务效益 (元，保留一位小数)

|  | 2009年 | 2010年 | 2011年 | 2012年 | 2013年 | 2014 |
| --- | --- | --- | --- | --- | --- | --- |
| 门诊均次费用 |  |  |  |  |  |  |
| 住院床日费用 |  |  |  |  |  |  |
| 家床日均费用 |  |  |  |  |  |  |

**五、居民总体满意度情况** (%，保留两位小数)

|  | 2009年 | 2010年 | 2011年 | 2012年 | 2013年 | 2014年 |
| --- | --- | --- | --- | --- | --- | --- |
| 总体满意度 |  |  |  |  |  |  |

**六、双向转诊情况 (次数)**

|  | 2009年 | 2010年 | 2011年 | 2012年 | 2013年 |
| --- | --- | --- | --- | --- | --- |
| 转诊到上级医院病例数 |  |  |  |  |  |
| 上级医院下转病例数 |  |  |  |  |  |

**七、信息化情况**

|  | 2009年 | 2010年 | 2011年 | 2012年 | 2013年 | 2014年 |
| --- | --- | --- | --- | --- | --- | --- |
| 电子健康档案建档率 |  |  |  |  |  |  |
| 实行中心内部信息化一体化管理 (有、无) |  |  |  |  |  |  |
| 和社区卫生服务站实行一体化信息管理 (有、无) |  |  |  |  |  |  |
| 医院-中心-站一体化信息化管理 (有、无) |  |  |  |  |  |  |
